# Age-related differences in clinical characteristics of invasive group G streptococcal infection: Comparison with group A and group B streptococcal infections

**DOI:** 10.1371/journal.pone.0211786

**Published:** 2019-03-07

**Authors:** Yoshihiro Fujiya, Kayoko Hayakawa, Yoshiaki Gu, Kei Yamamoto, Momoko Mawatari, Satoshi Kutsuna, Nozomi Takeshita, Yasuyuki Kato, Shuzo Kanagawa, Norio Ohmagari

**Affiliations:** 1 Disease Control and Prevention Center, National Center for Global Health and Medicine, Shinjuku-ku, Tokyo, Japan; 2 AMR Clinical Reference Center, National Center for Global Health and Medicine Hospital, Shinjuku-ku, Tokyo, Japan; Public Health England, UNITED KINGDOM

## Abstract

**Purpose:**

Invasive Group G streptococcal infection (iGGS) has increasingly been recognized as a cause of severe disease, mainly among elderly people with chronic illnesses. This study aimed to examine age-related differences in clinical characteristics of iGGS and describe its characteristics among very elderly individuals (≥80 years).

**Methods:**

Fifty-four iGGS patients for whom detailed clinical information was available were identified from 2002 to 2014 in a tertiary care hospital in Japan. iGGS (n = 54) was compared with invasive Group A (iGAS; n = 17) and B streptococcal infection patients (iGBS; n = 52) based on patient age.

**Results:**

The incidence of iGGS in our catchment area significantly increased during the study period. The prevalence of iGGS in the very elderly population was higher than that of iGAS or iGBS (p<0.001). Among iGGS patients, cardiovascular disease, chronic kidney disease, oxygen demand, and bacteremia with unknown focus of infection were more frequent in the very elderly population (p = 0.009, p = 0.02, p = 0.04, and p = 0.04, respectively). Altered mental status was present in half of iGGS patients aged ≥60 years (p = 0.03). In contrast, alcohol drinking and liver cirrhosis were significantly more frequent in patients with iGGS aged <60 years than in other age groups (p<0.001, p = 0.001, respectively). Levofloxacin resistance in GBS isolates was significantly more frequent among very elderly patients than among other age groups (p<0.001).

**Conclusions:**

The burden of iGGS has been increasing in our catchment area. Different iGGS-associated clinical characteristics were found in each age group. Unclear and atypical clinical manifestations and syndromes were likely to be observed in very elderly patients. Alcohol drinking and liver cirrhosis may contribute to iGGS even in patients aged <60 years. Understanding these age-related differences could be helpful for optimal diagnosis and treatment.

## Introduction

The beta-hemolytic streptococci (BHS) belonging to the Lancefield group A, B, and G (GAS, GBS, and GGS, respectively) are common pathogens for a wide spectrum of infectious diseases in humans. Invasive GAS and GBS infections (iGAS and iGBS, respectively), including bacteremia, pneumonia, arthritis, meningitis, necrotizing fasciitis, and streptococcal toxic shock syndrome (STSS), have been reported worldwide and are diseases with high fatality rates (typically 10–60% fatal) [1–6]. Recently, an increased number of cases of bacteremia due to GGS have been reported [7, 8]. GGS has been increasingly recognized as an important pathogen causing invasive infections. Invasive GGS infections (iGGS) occur most commonly in elderly patients and those with underlying diseases [9, 10]. Bacteremic cellulitis, bacteremia without focus, and bone and joint infections are the most common clinical manifestations among patients with iGGS [9–11]. Life-threatening diseases caused by GGS, such as necrotizing fasciitis, STSS, pneumonia, endocarditis, and others resembling those by GAS, have been reported recently, even though GGS is considered a less virulent pathogen than GAS [9, 11, 12]. Some studies indicate that the case fatality ratio of iGGS is as high as that of iGAS, and optimal clinical management is therefore essential [9, 11, 13].

iGGS seems to be associated with age and underlying medical conditions. Wajima et al. reported that the clinical syndromes of iGGS differ based on patients’ age; pneumonia, which is an atypical clinical syndrome in iGGS, was more frequent in the very elderly population ≥80 years, while septic arthritis and noncutaneous abscesses were more frequent in the young population [13]. Among elderly patients, nonspecific clinical expression of infection, which includes delirium, weakness, anorexia, and malaise, would be common instead of typical clinical manifestations in patients with infections, including fever, tachycardia, tachypnea, and organ-specific symptoms [14]. Some studies show that cardiovascular disease (CVD), diabetes, malignancy, and renal dysfunction are the most common underlying diseases in all age groups among patients with iGGS; however, specific age-related differences in underlying diseases are unknown [6, 9, 11, 13]. In particular, information on the clinical characteristics of very elderly patients (≥80 years) with iGGS are limited, despite the increase in the aging population in the world.

Here, we described the clinical characteristics of patients with invasive BHS infections (iBHS) in a tertiary care hospital over a 12-year period. Our aim was to determine age-related differences in the predisposing factors and clinical characteristics of iGGS and to describe its characteristics especially among very elderly individuals. Overall, we did observe age-related differences and have described in detail the characteristics associated with iGGS, especially among elderly patients.

## Materials and methods

### Study setting, patients, and definitions

This retrospective cohort study was conducted at the National Center for Global Health and Medicine (NCGM) hospital, a 781-bed tertiary care hospital in the Shinjuku district of Tokyo, Japan. Patients who developed invasive streptococcal infection due to BHS from January 2002 to January 2014 were included.

Invasive streptococcal infection was defined as the isolation of BHS from a normally sterile site: blood, cerebrospinal fluid (CSF), pleural fluid, ascites, and/or synovial fluid. The laboratory database was reviewed to identify patients with BHS isolation from the aforementioned sterile sites. Repeated positive cultures of the same organism within 30 days of the initial isolation were considered to be from a single episode based on previous reports [15, 16] and opinions from multiple infectious disease physicians in the research team. Data on demographic characteristics, as well as laboratory, microbiological, clinical, and patients’ outcome data, were retrieved from medical records. The patients aged 60–79 years were defined as elderly and those aged over 80 years were defined as very elderly.

Sepsis was diagnosed retrospectively if the patient met at least 2 quick Sequential Organ Failure Assessment (qSOFA) criteria: respiratory rate of ≥22 /min, altered mentation, or systolic blood pressure of ≤100 mmHg [17]. Patients in septic shock were identified with a clinical construct for sepsis with persisting hypotension requiring vasopressors and having a serum lactate level >2 mmol/L [17].

Skin and soft tissue infections (SSTIs) included cellulitis, infection on the site of pressure ulcer, subcutaneous abscess, myositis, and necrotizing fasciitis. Necrotizing fasciitis was limited to cases in which necrotic changes were pathologically confirmed in materials obtained during surgery. STSS was diagnosed according to the criteria proposed by the Working Group on Severe Streptococcal Infections in 1993 [18]. Outcome in our study was defined as all mortality after hospitalization due to any cause.

### Bacterial identification and antimicrobial susceptibility

Using standard methods [19], BHS isolates were identified by their morphological and growth characteristics. This included beta-hemolysis on sheep blood agar, Gram staining, and a negative catalase test. Serogroup was determined by the rapid agglutination test (Oxoid, Basingstoke, UK). The MicroScan WalkAway automated microbiology system (Siemens Healthcare Diagnostics, Tokyo, Japan) was used for strain characterization of the isolates. Antimicrobial susceptibility was tested using the microdilution method, and susceptibility was categorized according to the Clinical and Laboratory Standards Institute using the same automated system.

### Statistical analysis

The annual incidence of iGAS, iGBS, and iGGS was calculated using census data from the Shinjuku district population available for 2003 to 2013, because Shinjuku district is a medical catchment area for our hospital (https://www.city.shinjuku.lg.jp/kusei/index02_101.html). Age-adjusted incidence was calculated using the direct method and the Shinjuku district population in 2003 as the standard. Data were analyzed using SPSS PASW Statistics 18 (SPSS Inc., Chicago, IL, USA). Categorical variables were analyzed using the chi-square test or Fisher’s exact test, as appropriate. Continuous, non-parametric data were analyzed using the Mann-Whitney *U* test or Kruskal-Wallis test, as appropriate. Time trends were evaluated by dividing the data into an early cohort (2003–2007) and a late cohort (2009–2013), and calculating the mean incidence rate ratios (IRR) and a p-value. A two-tailed p- value <0.05 was considered statistically significant.

### Ethics statement

This study was approved prior to its commencement by the Human Research Ethics Committee of the National Center for Global Health and Medicine (NCGM-G-001572-00). All procedures performed in studies involving human participants were in accordance with the ethical standards of the institutional and/or national research committee and with the 1964 Helsinki declaration and its later amendments or comparable ethical standards. For this type of study (retrospective) formal consent from patients was not required.

## Results

**Fig 1:**
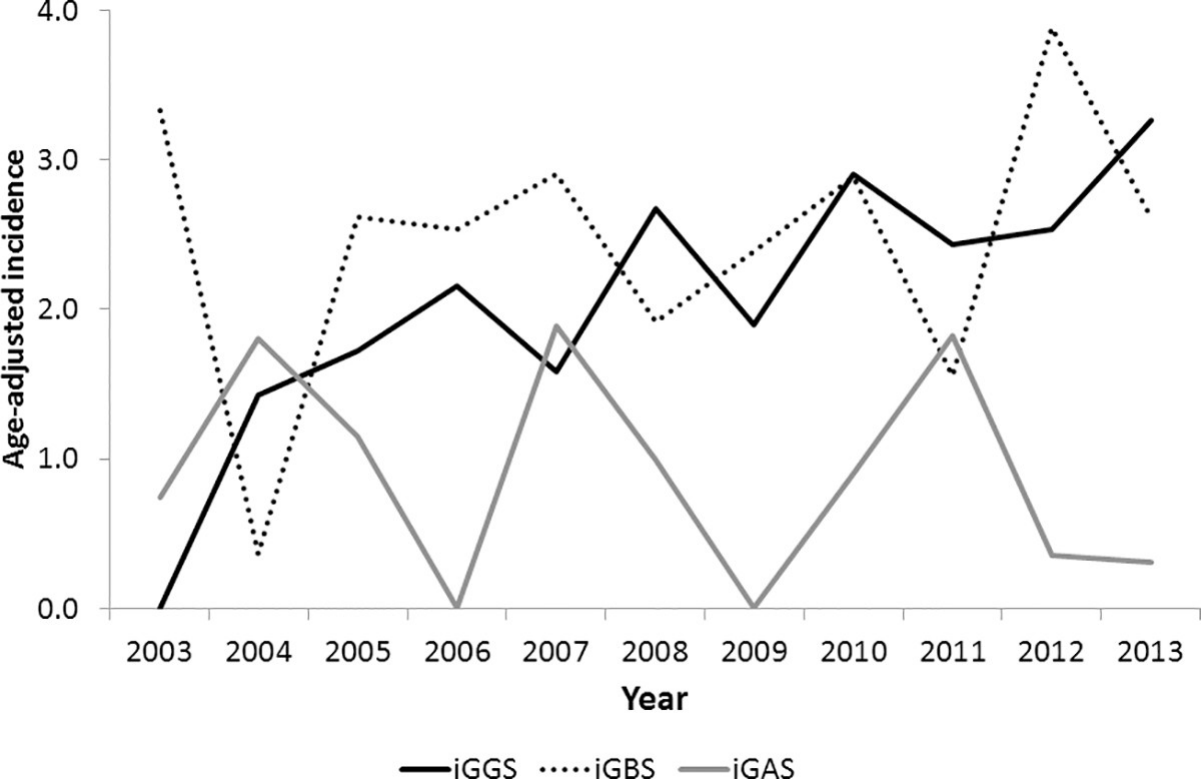
The incidence of invasive streptococcal infections per 100,000 inhabitants (2003–2013).

During the study period, 189 patients with invasive streptococcal infection were detected in the laboratory database; of these, there were 36 (19%), 83 (44%), and 70 (37%) patients with iGAS, iGBS, and iGGS, respectively. All patients had no recurrence and had only one episode of invasive infection. All GAS, GBS, and GGS were identified as *Streptococcus pyogenes*, *S*. *agalactiae*, and *S*. *dysgalactiae* subspecies *equisimilis*, respectively. All BHS were isolated from sterile site specimens; of these, 170 (90%) were from blood, 3 (2%) from CSF, 3 (2%) from pleural effusion, 6 (3%) from ascites, and 7 (4%) from synovial fluid. The overall age-adjusted incidences for iGAS, iGBS, and iGGS from 2003 to 2013 (95% confidence interval) were 0.9 (0.4–1.4), 2.5 (1.8–3.1), and 2.1 (1.5–2.7) per 100,000 inhabitants, respectively. Their annual trends are presented in Fig 1. There was a significant increase in iGGS incidence from the first to the last 5-year period (IRR 1.89, p = 0.02), with annual incidence peaking at 3.3/100,000 inhabitants in 2013. The iGAS incidence showed marked fluctuations over time, ranging from 0.0/100,000 (2006 and 2009) to 1.9/100,000 (2007). However, no statistically significant trend was observed (IRR 0.61, p = 0.40). The iGBS incidence remained steady from about 2.0/100,000 to 3.0/100,000, with no statistically significant trend (IRR 1.13, p = 0.92). During the most recent year (2013), iGGS was the dominant iBHS.

### Demographics and clinical information

Detailed demographic and clinical information was available for 54 (77%), 17 (47%), and 52 (63%) patients with iGGS, iGAS, and iGBS, respectively. These patients were included in further analyses.

**Fig 2:**
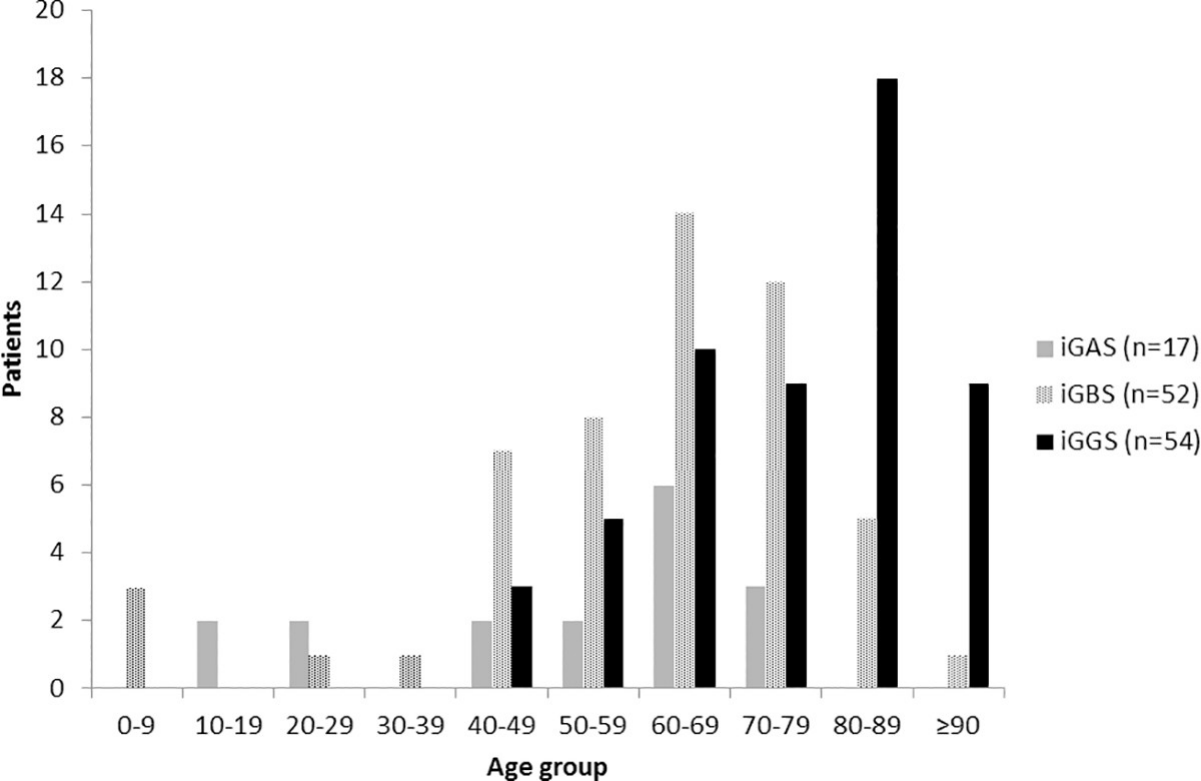
The number of patients with invasive streptococcal infections in each age group (2002–2013).

iGGS was most common in patients aged 80–89 years (Fig 2). The median age (in years) was 79.5 [interquartile range (IQR); 67–86], whereas that of iGAS and iGBS patients was 61 [IQR: 41–69] and 65.5 [IQR: 54–75], respectively (p<0.001). The proportions of patients ≥80 years differed significantly between iGGS, iGAS, and iGBS (p<0.001), and was highest in iGGS (iGGS 50% [n = 27], iGAS 0%, iGBS 11.5% [n = 6]).

Serogroup-related differences in clinical characteristics among iBHS in each age group are shown in Table 1. iGGS was more common in males among patients aged <80 years, compared with iGBS (p = 0.004). At symptom onset, half of all iGGS patients aged ≥60 years had altered mental status. There was no significant difference in the frequency of sepsis by serogroups. Chronic heart failure (CHF), chronic kidney disease (CKD), and neurologic disease were more likely to be present in very elderly patients; no significant difference was found when stratified by serogroup (p>0.10). Alcohol drinking and liver cirrhosis were more frequent among iGGS patients than iGAS and iGBS patients in the <60 years age group (p = 0.002 and p = 0.07, respectively).

**Table 1:** Characteristics of invasive Group G streptococcal infection as compared to invasive Group A and B streptococcal infections among different age groups.

Characteristics	Age <60				60≤ Age <80				80≤ Age		
	iGAS (n = 8)	iGBS (n = 20)	iGGS (n = 8)	p-value*	iGAS (n = 9)	iGBS (n = 26)	iGGS (n = 19)	p-value*	iGBS (n = 6)	iGGS (n = 27)	p-value*
**Demographics, No. (%)**											
Male	4 (50)	10 (50)	6 (75)	0.46	8 (89)	10 (39)	15 (79)	**0.004**	3 (50)	13 (48)	0.94
**Vital signs, No. (%)**											
Respiratory rate, ≥22 breaths/min	1 (20)	5 (56)	1 (33)	0.41	1 (14)	7 (37)	6 (40)	0.47	4 (80)	9 (53)	0.28
Systolic blood pressure, ≤100 mmHg	2 (29)	6 (40)	1 (13)	0.39	0 (0)	2 (9)	5 (26)	0.12	1 (17)	3 (12)	0.76
Altered mental status, GCS<15	0 (0)	1 (7)	0 (0)	0.57	1 (14)	5 (20)	10 (53)	**0.04**	5 (83)	13 (50)	0.14
Sepsis, qSOFA ≥2	0 (0)	3 (21)	0 (0)	0.19	1 (13)	3 (14)	7 (37)	0.16	4 (67)	8 (36)	0.18
**Underlying disease, No. (%)** ^a^											
Congestive heart failure	0 (0)	0 (0)	0 (0)	-	0 (0)	6 (23)	1 (5)	0.10	3 (50)	13 (48)	0.94
Chronic kidney disease	1 (13)	1 (5)	0 (0)	0.54	0 (0)	2 (8)	0 (0)	0.33	2 (33)	7 (26)	0.71
Neurologic disease	1 (13)	5 (25)	1 (13)	0.64	2 (22)	5 (19)	3 (16)	0.91	3 (50)	8 (30)	0.34
Cirrhosis	2 (25)	2 (10)	4 (50)	0.07	0 (0)	3 (12)	3 (16)	0.46	0 (0)	0 (0)	-
Alcohol drinking	2 (25)	2 (10)	6 (75)	**0.002**	2 (22)	1 (4)	4 (21)	0.16	0 (0)	0 (0)	-
**Clinical syndrome, No. (%)** ^a^											
Bacteremia without focus	1 (13)	6 (30)	0 (0)	0.17	1 (11)	6 (23)	1 (5)	0.24	2 (33)	8 (30)	0.86
Skin and soft tissue infections ^b^	6 (75)	5 (25)	5 (63)	**0.03**	6 (67)	12 (46)	12 (63)	0.40	1 (17)	15 (56)	0.09
upper extremities ^c^	1 (17)	0 (0)	1 (20)	0.59	2 (33)	0 (0)	0 (0)	**0.01**	0 (0)	1 (7)	0.79
lower extremities ^c^	3 (50)	5 (100)	4 (80)	0.16	2 (33)	7 (58)	11 (92)	**0.03**	1 (100)	12 (80)	0.62
Septic arthritis	3 (38)	0 (0)	3 (38)	**0.01**	2 (22)	2 (8)	4 (21)	0.36	0 (0)	5 (19)	0.25
Pneumonia	0 (0)	0 (0)	1 (13)	0.17	1 (11)	1 (4)	0 (0)	0.35	3 (50)	0 (0)	**<0.001**
**Outcome, No.(%)**											
Died in hospital	0 (0)	0 (0)	0 (0)	-	0 (0)	2 (8)	0 (0)	0.33	1 (17)	0 (0)	**0.03**

Abbreviations: iGAS, invasive Group A streptococcal infection; iGBS, invasive Group B streptococcal infection; iGGS, invasive Group G streptococcal infection; GCS, Glasgow Coma Scale; qSOFA, quick Sequential Organ Failure Assessment

*Categorical variables were analyzed using the chi-square test or Fisher exact test and continuous, non-parametric data were analyzed using the Mann-Whitney U test or Kruskal-Wallis test across 3 groups, which were iGAS, iGBS, and iGGS patient groups, as appropriate. A p-value <0.05 was statistically significant, and indicated in bold and highlight

^a^ A patient may have had one or more underlying diseases or clinical syndromes

^b^ Includes cellulitis, infection on the site of pressure ulcer, subcutaneous abscess, myositis, and necrotizing fasciitis

^c^ The denominator is the number of patients with all SSTI in each age group and serotype, and SSTI may be present on one or more sites of body

Regarding clinical syndromes (Table 1), SSTIs were more frequently observed in iGGS and iGAS patients than iGBS patients. SSTIs in iBHS patients were frequently found on their lower extremities, especially in iGGS patients. Septic arthritis was more likely to be observed in iGGS and iGAS patients than iGBS patients. In the ≥80 years age group, pneumonia was significantly more common in iGBS patients than in iGGS patients (n = 3 [50%] vs. n = 0, p<0.001). No fatalities occurred among iGGS and iGAS patients during the study period, whereas 3 (6%) iGBS patients died. The three patients that died of iGBS had received an antibiotic to which the bacteria were susceptible. Two of these patients had pneumonia with septic shock and the other patient had severe liver cirrhosis and bacteremia.

Given its prevalence in the very elderly population, we analyzed age-related differences in characteristics of iGGS patients (Table 2). A higher proportion of patients aged <80 years were male (78%) compared to those aged ≥80 years (48%) (p = 0.07). Half of all iGGS patients aged ≥60 years had altered mental status, whereas none aged <60 years did (p = 0.007). Fifteen (37%) patients aged ≥60 years had sepsis compared to none aged <60 years (p = 0.04). In the acute phase, patients aged ≥80 years were more likely to need oxygen supply (p = 0.04). All but one patient aged <60 years with iGGS had one or more underlying diseases. CVD, especially CHF, and CKD were more frequent in the elderly aged ≥80 years (p = 0.009, p = 0.001, and p = 0.02, respectively). Alcohol drinking and liver cirrhosis were reported in more than half of patients <60 years, but none in the ≥80 years (p<0.001 and p = 0.001, respectively). There were no differences in the frequencies of SSTI, including cellulitis, and septic arthritis among the age groups; however, bacteremia with unknown focus of infection were more likely to be observed in patients ≥80 years (p = 0.04).

**Table 2:** Characteristics of invasive Group G streptococcal infection by age group.

Characteristics	No. (%) of patients by age group			Total (n = 54)	p-value*
	<60 y (n = 8)	60–79 y (n = 19)	≥80 y (n = 27)		
**Demographics**					
Male, No. (%)	6 (75)	15 (79)	13 (48)	34 (63)	0.07
**Clinical manifestations** ^a^					
Vital signs, No. (%)					
Respiratory rate, ≥22 breaths/min	1 (33)	6 (40)	9 (53)	16 (30)	0.69
Systolic blood pressure, ≤100 mmHg	1 (13)	5 (26)	3 (12)	9 (17)	0.43
Altered mental status, GCS<15	0 (0)	10 (53)	13 (50)	23 (43)	**0.03**
Severity, No. (%)					
Sepsis, qSOFA ≥2	0 (0)	7 (37)	8 (36)	15 (28)	0.12
Interventions, No. (%)					
Oxygen supply	0 (0)	1 (5)	7 (30)	8 (15)	**0.04**
**Underlying disease, No. (%)** ^b^					
None	1 (13)	0 (0)	0 (0)	1 (2)	0.05
Cardiovascular disease	2 (25)	7 (37)	20 (74)	29 (54)	**0.009**
Congestive heart failure	0 (0)	1 (5)	13 (48)	14 (26)	**0.001**
Chronic kidney disease	0 (0)	0 (0)	7 (26)	7 (13)	**0.02**
Cirrhosis	4 (50)	3 (16)	0 (0)	7 (13)	**0.001**
Alcohol drinking	6 (75)	4 (21)	0 (0)	10 (19)	**<0.001**
**Clinical syndrome, No. (%)** ^b^					
Bacteremia without focus	0 (0)	1 (5)	8 (30)	9 (17)	**0.04**
All skin and soft tissue infections (SSTI)	5 (63)	12 (63)	15 (56)	32 (59)	0.86
Septic arthritis	3 (38)	4 (21)	5 (19)	12 (22)	0.52

Abbreviations: GGS, Group G Streptococcus; GCS, Glasgow Coma scale; qSOFA, quick Sequential Organ Failure Assessment

* Chi-square test was performed across 3 groups, which were the <60, 60–75, and ≥80 years age group. A p-value <0.05 was statistically significant, and indicated in bold and highlight.

^a^ The percentage is of patients for whom data were available, i.e., excluding the missing cases

^b^ A patient may have had one or more underlying diseases or clinical syndromes

### Antimicrobial resistance

We analyzed antimicrobial resistance of 54 (77%) GGS, 17 (47%) GAS, and 52 (63%) GBS isolates (Table 3). All GGS isolates were susceptible to beta-lactam agents and vancomycin. Seven (13%) GGS isolates were resistant to erythromycin and three (6%) were resistant to clindamycin. Compared with GAS and GBS isolates, there were no significant differences in erythromycin- and clindamycin-resistance (p = 0.67 and 0.23, respectively). All GGS isolates were susceptible to levofloxacin, whereas one (6%) GAS and 12 (23%) GBS isolates were resistant (p<0.001). In GBS isolates, the proportions with resistance to levofloxacin differed between age groups: 5/6 (83%) in ≥80 years, 6/26 (23%) in 60–79 years, and 1/20 (5%) in <60 years (p<0.001).

**Table 3:** Antimicrobial resistance in Group A, B, and G streptococci isolated from patients with invasive infections, No. (%).

Antimicrobial resistance	GAS (n = 17)	GBS (n = 52)	GGS (n = 54)	P-value*
Penicillin-resistance	0 (0)	0 (0)	0 (0)	-
Ampicillin-resistance	0 (0)	0 (0)	0 (0)	-
Cefotaxime-resistance	0 (0)	0 (0)	0 (0)	-
Erythromycin-resistance	3 (18)	10 (19)	7 (13)	0.67
Clindamycin-resistance	0 (0)	6 (12)	3 (6)	0.23
Vancomycin-resistance	0 (0)	0 (0)	0 (0)	-
Levofloxacin-resistance	1 (6)	12 (23)	0 (0)	**<0.001**
Total**	4 (24)	23 (44)	7 (13)	

Abbreviations: GAS, Group A streptococcus; GBS, Group B streptococcus; GGS, Group G streptococcus

* Chi-square test was performed. A p- value <0.05 was statistically significant and indicated in bold and highlight.

** This indicates the total number of the isolates resistant to one or more antimicrobial agents.

## Discussion

The purpose of this study was to show age-related differences in clinical characteristics of invasive streptococcal infection, particularly iGGS. Over 12 years, iGGS was dominant in very elderly patients aged ≥80 years, and the incidence of iGGS had been increasing. CVD and CKD as comorbidities, unknown focus of infection, and atypical clinical manifestations, such as altered mental status, were frequent among very elderly patients with iGGS.

Population-based incidence of iBHS has been reported from many countries [1–5, 8, 9, 11, 16, 20, 21]. However, as only STSS is a notifiable disease in Japan, few reports have been published and are insufficient to understand the burden of iBHS in Japan. Takahashi et al. and Wajima et al. reported the number of iBHS patients in Japan from nationwide sentinel surveillance in 1-year and 3-year surveys, respectively; however, until now, the incidence and long-term trend in Japan remained unknown [6, 13]. The overall incidence of iGAS in our study (0.9/100,000) was much lower than many European countries and the United States (2-4/100,000) [1–3, 20]. The overall incidence of iGBS (2.5/100,000) in our study was similar to those in some European countries (2-3/100,000), whereas a higher rate was observed in the United States (7.3/100,000) [4, 5, 20]. The overall incidence of iGGS (2.1/100,000) was lower than that in Denmark (2.6/100,000) and the United States (3.2/100,000) [11, 20]. The burden of iGGS had been significantly increasing in our medical catchment area and iGGS became dominant in the most recent year (2013). Although iGAS or iGBS have been dominant in European countries and the United States, an increase in iGGS has been reported, especially in Northern Europe [1, 5, 11, 16, 20–22]. Of note, these differences among different geographical regions should be carefully interpreted as this might be due to differences in reporting systems (e.g., denominator, time period, and surveillance methods). In addition, the incidence may not necessarily be representative in our catchment area because some iBHS patients might have received treatment in other hospitals. A likely explanation for the increase in the incidence of iGGS in our study is the aging population. Some reports have suggested that immunosenescence associated with aging and a concomitant increase in comorbidity contributes to the risk of iGGS [6, 13, 16, 23]. Population data from the Shinjuku district where NCGM is located indicates that the total population in Shinjuku increased by 7% from 2002 to 2013; however, the population aged ≥65 years and ≥80 years increased by 21% and 46%, respectively over the same period (https://www.city.shinjuku.lg.jp/kusei/index02_101.html). We assume that the rapid increase in the aged population in the Shinjuku area could have influenced the incidence of iGGS, and a further increase in iGGS could be expected in the future. A national surveillance system including data that is comparable to those from other countries is needed in Japan to monitor the burden of iBHS continuously.

Several studies have reported clinical syndromes by age groups for each invasive streptococcal infection. Among iGAS patients, pneumonia, bacteremia, or SSTI were more frequent in the elderly population [1–3]. Among non-pregnant adult patients with iGBS, bacteremia, pneumonia, or urinary tract infection were more common in patients aged ≥65 years [5]. Among iGGS patients, SSTI, and bacteremia with no focus of infection are common in elderly individuals [9]. In particular, pneumonia occurs more frequently in those aged ≥80 years, as shown by a Japanese study [13]. In our study, almost all patients with iGGS aged <80 years had identified foci of infection; however, one third of those aged ≥80 years had bacteremia with no focus of infection. In addition, altered mental status and the necessity of oxygen supply were more frequently observed in very elderly patients with iGGS, even in those without pneumonia, than in patients with other iBHS. Some previous reports indicate that the clinical manifestations in infected elderly patients are likely to be too imperceptible to be recognized and atypical clinical expressions, including a change in mental status or a decline in physical functional status, are common in the elderly with sepsis [14, 24]. Therefore, it may be challenging to diagnose iGGS in very elderly patients. Careful evaluation for subtle and atypical signs of sepsis (such as altered mental status and demand of oxygen supply without pneumonia) combined with appropriate culture tests, and close follow-up of the clinical course will aid the early diagnosis of iGGS in very elderly patients.

We also found that underlying diseases in patients with iGGS are likely to differ by age groups. Underlying diseases associated with iGGS such as CKD, diabetes mellitus, malignancy, liver or renal dysfunction, chronic skin disease, and obesity have been reported [6, 9, 11, 13, 25]; however, age-related differences were unknown. CVD and CKD were more commonly observed in very elderly patients with iGGS than in those with other iBHS. This may be due to the increased prevalence of CVD and CKD with age [26, 27]. In contrast, alcohol drinking and liver cirrhosis were significantly more frequent in patients with iGGS aged <60 years than other age groups. This is consistent with studies that suggest that alcohol drinking and liver cirrhosis are generally more common in patients in their 50s [28, 29] than in older adults. However, there were no significant age-related differences in comorbidities in patients with iGAS and iGBS. Among all iBHS patients aged <60 years, alcohol drinking and liver cirrhosis were observed more frequently in patients with iGGS. One possible explanation for this is that alcohol drinking and liver cirrhosis could lead to an immunosuppressed state by affecting the immune system. Alcohol and liver cirrhosis alter the production and function of neutrophils, macrophages, monocytes, and T and B lymphocytes and then lead to the dysfunction of the innate and adaptive immune system, such as immunosenescence due to aging [23, 30–32]. In addition, alcohol and liver cirrhosis impair epithelial cells linking the mucosa of the gut, leading to increased gut permeability [31, 33, 34]. This mucosal permeability changes and local immunological alterations in the gut allow the translocation of bacteria and/or bacteria-derived products into the blood stream [35]. GGS is commonly found in the pharynx, gastrointestinal and female genital tracts, and in the skin in humans [9]. We assume that the translocation from the gut might be one mechanism of iGGS among patients with alcohol use and liver cirrhosis.

All isolates, including GGS, were susceptible to beta-lactam agents in this study. Penicillin-resistant GGS has been very rare so far, but some cases were reported in Denmark and Japan [36, 37]. Our study identified macrolides-, clindamycin-, and fluoroquinolone-resistant BHS. As macrolide-resistant isolates have not been routinely tested for inducible clindamycin resistance in our hospital, there might have been more clindamycin-resistant BHS. In particular, levofloxacin resistance in GBS isolates was significantly more frequent among very elderly patients than among other age groups. Resistance to these agents has been shown in many countries; however, few reports have compared the frequency of antimicrobial resistance by age groups in BHS causing invasive infection [4, 11, 38, 39]. We assume that the frequency of antimicrobial use among the elderly population might affect this age-related difference. Yamasaki et al. indicated the high proportion of total antimicrobial use and frequent use of oral fluoroquinolones among the elderly group aged >65 years in Japan [40]. Clinicians would need to note this trend of antimicrobial resistance in BHS isolates.

We acknowledge several limitations in this study. As this was conducted in a single institution, the results may have some regional and/or institutional biases, and may not necessarily be representative of the larger population. Due to the retrospective design of the study, there were missing data, mainly owing to the use of paper-based medical records until 2010. Therefore, we could include only the patients with sufficient available clinical information.

This study provides insights into age-related differences in predisposing factors and clinical manifestations in iGGS, as well as antimicrobial resistance in iGBS. According to the World Population Prospects (2017), the proportion of the very elderly population aged ≥80 years, likely to have any chronic illness, will be expected to increase worldwide [41]. It is possible that the burden of iGGS will continue to increase. We believe understanding these clinical characteristics in very elderly patients would be useful for optimal clinical management. Moreover, clinicians may need to consider iGGS even in patients presenting with sepsis aged <60 years if they have history of alcohol use and liver cirrhosis. However, further prospective and large-scale studies are warranted to clarify age-related differences in clinical characteristics in invasive streptococcal infections.

## Supporting information

S1 Table Characteristics of invasive Group G streptococcal infection as compared to invasive Group A and B streptococcal infections among different age groups. (XLSX)

S1 Dataset Dataset of iGAS, iGBS, and iGGS patients. (XLS)
